# Improving Models to Predict Care Utilization Using Machine Learning: Retrospective Observational Study

**DOI:** 10.2196/92820

**Published:** 2026-06-26

**Authors:** Christopher Kitchen, Talan Zhang, Klaus Lemke, Chintan Pandya, Hadi Kharrazi, Jonathan P Weiner

**Affiliations:** 1 Department of Health Policy and Management, Bloomberg School of Public Health, Johns Hopkins University, 2024 E Monument Street, Baltimore, MD, United States, 1 3015310011; 2 Center for Biomedical Informatics and Data Science, School of Medicine, Johns Hopkins University, Baltimore, MD, United States

**Keywords:** medical informatics, risk stratification, clinical decision support, machine learning, public health informatics

## Abstract

**Background:**

The use of artificial intelligence and machine learning (ML) tools is now common in the advancement of health care services and clinical risk estimation. Legacy systems make use of highly informative feature sets developed from years of clinical expertise and research to estimate different outcomes, but only recently have they been tested against novel statistical approaches. One such system, the Johns Hopkins Adjusted Clinical Group (ACG) System, is a long-standing and widely used approach to categorizing clinical risk factors, and it is amenable to ML techniques.

**Objective:**

This study aims to test the ACG System using a contrasted area under the receiver operating characteristic (AUROC) and *F*_1_ classification optimization strategy and compare its performance against traditional logistic regression methods. Assuming that selected ML algorithms can be tuned to enhance overall measures of performance, this would strengthen arguments for incorporating them into ACG-related workflows.

**Methods:**

Using a retrospective observational design, prospective year estimates of all-cause hospitalization and elevated total cost were modeled using a cross-validation framework. Patients with elevated costs were identified as those falling above the 95th percentile of total amounts billed, including pharmacy costs. Hyperparameter settings for XGBoost (Extreme Gradient Boosting), random forest, and elastic net were determined using average cross-validated performances for *F*_1_ and AUROC in a grid search aimed at maximizing either statistic. Additional iterated cross-validation was used to compare point-estimated average AUROC and *F*_1_-scores between models, further decomposed by sensitivity, positive predictive value, and *F*-beta statistics.

**Results:**

There were 350,463 patients selected in 2019 from the Johns Hopkins Health System. Model features identified by the ACG System for predicting prospective year hospitalization and total cost were included in these analyses. Findings suggest small but statistically significant improvements in cross-validated AUROC and *F*_1_-scores over logistic regression, using either optimization strategy and XGBoost. Logistic models achieved average receiver operating characteristic values of 0.886 and 0.841 for cost and hospitalization, respectively, whereas XGBoost achieved 0.891 and 0.849, respectively. *F*_1_ optimization yielded similar findings, with logistic models achieving 0.367 and 0.341 on average for hospitalization and cost, respectively, but XGBoost exceeded values for cost but not for hospitalization (0.411 and 0.328, respectively).

**Conclusions:**

The clinical implications of these findings and the effect of class imbalance on model calibration are explored, along with the limitations of these data and approach. The core finding is that logistic regression remains very well-suited to these tasks, especially in situations where the efficiency or interpretability of models is critical. Under conditions of imbalance, regressions tended to yield high-precision estimates for the outnumbered class. Nevertheless, the findings also underscore a diversity of suitable models depending on clinical use cases, each having its own tradeoffs for evaluating performance. As such, health systems must clearly identify the needs and expectations of a model before calibrating one for use.

## Introduction

### Background

Artificial intelligence (AI) and machine learning (ML) have been applied to risk stratification and predictive modeling within health care settings for several decades [1,2]. Some of the earliest examples include rule-based systems and conditional probability approaches to aid decision-making [2,3]. Today, health risks are frequently calculated through statistical modeling that accounts for demographic information, clinical comorbidities, laboratory findings, pharmacy records, and other important clinical or health care delivery characteristics. Health insurance claims represent a rich source of information for this purpose [4]. Recent high-profile advances in AI and ML have promulgated the narrative that newer, more complicated approaches are superior at modeling risk compared to regression techniques. ML tools are known to have certain advantages over regression, in part because they rely on fewer assumptions and might incorporate nonlinear associations with outcomes [5-7]. Additionally, many ML approaches allow hyperparameter tuning to optimize metrics for specific use cases, increasing sensitivity without compromising precision. Although research is still ongoing, many recent comparative analyses suggest that advanced ML may not always be superior or at least that improvements to model performance have been modest compared to traditional approaches [8-12].

In the real-world context of health care delivery, it is critical to consider the operational benefits and costs of using different quantitative risk models. Specific operational issues must be understood and assessed when attempting to evaluate statistical models [13]. Prediction of mortality and risk of hospitalization, for example, is challenging because these tend to constitute imbalanced class problems. Estimating health-related costs, as an overall measure of utilization and patient risk, tends to result in extremely skewed response distributions, making continuous outcomes a similarly nuanced concern [14-16]. Many real-world tasks might, therefore, be better understood as supervised anomaly detection and outlier estimation tasks, not just ones involving typical classification and regression performance estimates [17,18]. Clinicians care more about precision and residuals among high-risk individuals than among those least at risk.

This concern has been expressed by health care researchers and might be remedied through combinations of parameter tuning and cohort sampling techniques [16,19]. The generalizability of such approaches is problematic, however, leading to faulty conclusions about model performance, often due to sample bias [20-22]. Researchers also frequently report sensitivity, specificity, and C-statistics but fail to acknowledge practical limitations of these evaluation metrics or perform analyses with cost weighting applied from the clinical context. This may mask some critical differences between models, especially when applied to costly clinical decision support programs [13,22-24]. This criticism is especially important for imbalanced data and has been raised as a justification for relying on the increasingly popular *F*-beta statistic: the weighted harmonic average of precision and recall.

To demonstrate the potential for added predictive value in ML over traditional techniques, we selected a high-performance risk model for clinical risk estimation, the Johns Hopkins Adjusted Clinical Group (ACG) System. The ACG System incorporates clinical risk groupings for multiple diagnostic, care utilization, pharmacy, and demographic characteristics into a sequence of well-validated binary features. These features then form a basis for multiple predictive models published with the software, predicting expected costs, probability of hospitalization, and readmissions with multiple calibrations available for different age groups and lines of business [25]. Because these models are derived from extensive research and clinical expertise, we consider the ACG System’s modeling features to be among the most robust and detailed commercially available tools.

### Objectives

The aim of this work is to compare the relative performance of regression and decision tree ensemble methods using the ACG System’s sets of predictive features, evaluating 2 clinical outcomes: all-cause inpatient hospitalization and elevated cost. Though these outcomes specifically correspond to patient care utilization, they are also informative as general indicators of clinical risk [11,14,16]. We explore different optimization strategies and performance statistics to interpret a diversity of findings, addressing the common practice of locally calibrating models for user data. Such recalibration is burdensome to health systems, and ML approaches to estimation might be hard to adopt if local calibration also requires an intensive optimization search for each setting.

## Methods

### Participants and Setting

Patients from the Johns Hopkins Health Plans (JHHP) for the years 2019 to 2020 were considered for this retrospective cohort design. JHHP is a managed care organization that is part of the Johns Hopkins Health System. JHHP fully insures care for patients receiving treatment at any location (not just from the Johns Hopkins Health System). Our data were drawn from three lines of business: (1) the Employee Health Program, (2) Medicare Advantage, and (3) the Priority Partners Medicaid contracting health plan. Patients were selected if they had at least 1 month of enrollment in a single line of business during both the concurrent year (2019) and the prospective year of analysis (2020). A total of 350,463 such patients were identified.

Organization and quality of this analysis relied on the STROBE (Strengthening the Reporting of Observational Studies in Epidemiology) guidelines for reporting, which were completed where appropriate for the aims of this work (Checklist 1).

### Ethical Considerations

This work has been reviewed by the institutional review board at Johns Hopkins Bloomberg School of Public Health, approved as exempt (IRB00005784), and determined to be exempt from requiring patient consent. All data have been deidentified for secondary use in observational research, and patients could not be contacted for consent to participate as a result.

### Variable Definitions

Health claims were aggregated into an annual summary for each patient-year using the ACG System (version 13.0), published by Johns Hopkins Healthcare Solutions. The patient summary file is used to understand the characteristics of a cohort, such as the number of patients with a hospital admission, the number of chronic conditions, and patient demographics.

No patients were found to have missing data among the ACG System variables used for modeling as part of this research. Binary features are generally regarded as absent when not explicitly coded for in claims. For example, a claim identifying a 50-year-old woman with 2 concurrent-year emergency room visits would have a positive value for modeling features acg_4554, female, and edpat_2.

The analytical files consisting of ACG predictive features (model markers) were also developed for each annualized outcome: all-cause inpatient hospitalization and elevated total health care cost, defined as above the 95th percentile for billed amounts for each patient. Additional binary features included for these outcome-specific models are multiple ranges in age, concurrent year cost ranges associated with care, counts of concurrent year emergency room visits, hospitalizations, and outpatient encounters, clustered Resource Utilization Band, diagnosis-based morbidity risk (eg, expanded diagnosis cluster and adjusted clinical group), and pharmacy-based morbidity risk group. Specific description labels and the data dictionary for each feature can be found in the ACG System manual and are restricted to subscribers of these tools. There were a total of 268 features included in the ACG prospective year hospitalization models (all ages) and 251 for prospective year cost (all ages).

### Model Training and Selection

For all model types, concurrent-year observations (ie, 2019 annualized markers) are used to predict the likelihood of all-cause hospitalization or high cost in 1 year. Three ML approaches were explored in a cross-validated hyperparameter tuning framework, along with 3 regression-based methods commonly used in our risk stratification tasks. ML techniques included elastic net, random forest, and Extreme Gradient Boosting (XGBoost). Algorithms were selected on the basis of interpretability, scalability, and ability to handle high-dimensional data (ie, hundreds of features) [13,26,27]. Added parsimony is often a stated reason for using several techniques (eg, random forest, elastic net, and regularized models), but improved prediction is also frequently observed with reduced model variance [12,14,28,29].

The 3 non-ML regression models include logistic regression, least absolute shrinkage and selection operator (LASSO) regression, and a “reduced” multivariate regression consisting of just the set of inputs selected through LASSO and recalibrated without a regularization term. LASSO is a regularization technique that shrinks highly collinear coefficients to zero, dropping them from the model and weighing the remaining independent effects. All models were fitted using the R programming language (version 4.0.2; R Foundation for Statistical Computing) with relevant packages for each model type, including “glmnet,” “xgboost,” “randomForest,” and additional model evaluation tasks using “pROC” [30-32].

### Evaluation Framework or Parameter Settings

Evaluation metrics commonly used in ML tasks include area under the receiver operating characteristic (AUROC) and point-estimated precision and recall. A criticism of using AUROC in imbalanced data is that it tends to overestimate performance for models with poor sensitivity. This is due to the high count of negative cases, which arbitrarily reduces the false positive rate and subsequently inflates both specificity and AUROC [19,21]. Researchers have also made use of *F*_1_-scores to focus on the calibration of errors for just positive cases in imbalanced data [13,15]. The *F*_1_ is part of a family of *F*-beta scores that weigh precision and recall differentially, mimicking the cost-benefit exchange for dissimilar use cases. The *F*_1_ fixes this balance where precision (positive predictive value) and recall (sensitivity) are weighted equally, and it represents the harmonic average of the two.

Highly disparate model performances were possible through the tuning of hyperparameters. Settings were evaluated such that 2 optimization strategies were distinguished, maximizing either AUROC or *F*_1_. Parameters yielding the best average result in a 5-fold cross-validation for each setting were assigned through a small-scale grid search (Table S1 in Multimedia Appendix 1).

### Statistical Analyses

Cohort characteristics were explored through a stratified concurrent-year line of business and Patient Need Group (PNG), illustrating features including age, sex, number of chronic conditions, number of active ingredients, total cost, pharmacy cost, and utilization of certain points-of-care. PNG is an ACG classification of patients into 11 expected health care need groups using an index of comorbidity and patient care utilization [33].

Model performances are evaluated using 20-iteration 5-fold cross-validation, with conditions for optimized AUROC or *F*_1_. Average estimated performance is further characterized by respective 95% CIs for AUROC, *F*_1_, sensitivity, and positive predictive value (PPV). Wherever a cross-validated point estimate lies outside the 95% CI of a comparison model, we consider the average performance to be significantly different.

Due to the volume of models in this cross-validated framework, it was not possible to inspect all features for meaningful associations with outcomes or differences between model types. Furthermore, decision tree ensembles do not have a method for identifying discrete effects, akin to log odds or null hypothesis testing. Instead, we interpret features by variable importance across all applicable model types. This approach involves transforming the absolute value of coefficients from regressions and the average entropy reduction for decision tree models to a scale between 0 and 1. To document model parsimony, the number of features used for each model is presented with the main findings, and the average importance of the top 20 attributes for prospective-year hospitalization and elevated total cost is explored as a secondary analysis.

## Results

### Patient Characteristics

The selected cohort is a large, diverse sample that generally reflects insured patients from the Washington-Baltimore metropolitan area between 2019 and 2020 (Table 1). The average patient age was 24.1 (SD 20) years, with most of the sample belonging to the Priority Partners line of business (285,817/350,463, 81.6%; Table 1). The sample was majority female (193,984/350,463, 55.4%), and 65.2% (228,341/350,463) had a low-need or low-complexity PNG designation (Table S2 in Multimedia Appendix 1). Nearly half of the patients did not have a recorded race or ethnicity designation (47.6%). This met our expectations; however, as documentation of race or ethnicity is known to be sparse for many administrative claims databases [34].

Of 350,463 patients, 164,536 (46.9%) patients had 1 or more ACG-defined chronic conditions, and 240,528 patients (68.6%) took 1 or more active ingredients as part of a medication regimen. Across all patients, 6.3% (22,075/350,463) had a hospitalization in 2019. These hospitalizations tended to occur among those in the multimorbidity, high-complexity, and frailty PNGs; 48.3% (6911/14,321) and 47.2% (468/992) had concurrent-year hospitalizations, respectively; while 41.6% (5959/14,321) and 49.7% (493/992) were among the 95th percentile of patients with elevated concurrent-year health care costs.

**Table 1.** Patient characteristics by line of business.

	Line of business			Total sample
Variable	Employee health plan	Medicare advantage	Priority partners	
Total patients, N (%)	48,630 (100)	16,016 (100)	285,817 (100)	350,463 (100)
Average age (SD), y	33.5 (18.3)	69.8 (8.9)	20.0 (16.8)	24.1 (20.0)
Age, y, n (%)				
0‐17	10,942 (22.5)	0 (0)	159,757 (55.9)	170,699 (48.7)
18‐34	14,589 (30.0)	80 (0.5)	66,140 (23.1)	80,809 (23.1)
35‐64	21,850 (44.9)	2883 (18.0)	59,920 (21.0)	84,653 (24.2)
≥65	1249 (2.6)	13,053 (81.5)	0 (0)	14,302 (4.1)
Female, n (%)	28,083 (57.7)	9363 (58.5)	156,538 (54.8)	193,984 (55.4)
Male, n (%)	20,547 (42.3)	6653 (41.5)	129,279 (45.2)	156,479 (44.6)
Count of patients with 1 or more chronic conditions, n (%)	24,977 (51.4)	14,813 (92.5)	124,746 (43.6)	164,536 (46.9)
Count of patients with 1 or more active ingredients, n (%)	29,706 (61.1)	14,878 (92.9)	195,944 (68.6)	240,528 (68.6)
Cost >95th percentile, n (%)	4105 (8.4)	2340 (14.6)	11,079 (3.9)	17,524 (5.0)
Count of patients with 1 or more inpatient visit, n (%)	2103 (4.3)	1790 (11.2)	18,182 (6.4)	22,075 (6.3)

### Model Evaluation

**Figure 1.**
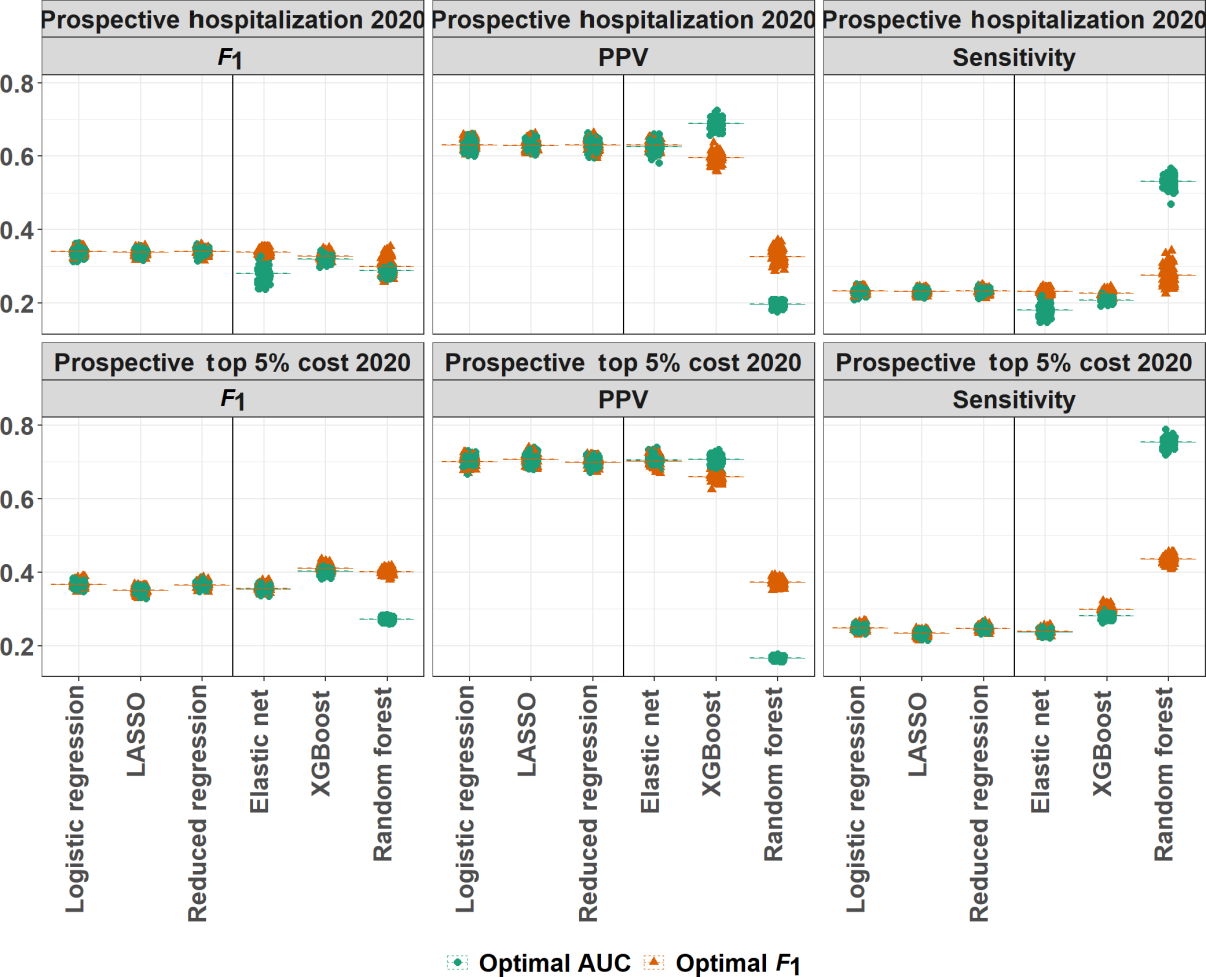
Cross-validated model performance by outcome (rows) and classification metric (columns), assuming a threshold of *P*(*x*)>.5. Horizontal dashed lines depict average performance for each trace. AUC: area under the curve; LASSO: least absolute shrinkage and selection operator; PPV: positive predictive value; XGBoost: Extreme Gradient Boosting.

Table 2 details the average cross-validated model performance with respect to each optimization objective and outcome. When *F*_1_ is maximized, XGBoost was found to have the highest average performance for identifying patients above the 95th percentile in prospective-year total health care cost (*F*_1_-score=0.411, 95% CI 0.409‐0.412), followed by random forest (*F*_1_-score=0.401, 95% CI 0.400‐0.403) and logistic regression (*F*_1_-score=0.367, 95% CI 0.366‐0.369). Both XGBoost and random forest models were significantly different in *F*_1_ performance compared to their respective logistic regression counterparts (Table S3 in Multimedia Appendix 1). The improvement in *F*_1_ over logistic regression was attributed to greater average sensitivity (Figure 1). The XGBoost model correctly identified 29.8% of patients in the prospective-year 95th percentile of total cost, compared with 24.8% for logistic regression. The same was not true for prospective-year hospitalized patients, where logistic regression was identified as the best-performing model for *F*_1_ optimization (*F*_1_-score=0.341, 95% CI 0.339‐0.342), followed by the remaining regression-based models, although equal sensitivity was roughly noted across all models. Average PPV was consistently and significantly lower than that of regressions for both XGBoost and random forest on their respective tasks (Table S3 in Multimedia Appendix 1).

**Table 2.** Ranked models by outcome, optimization, and cross-validated average model performance for binary outcomes.

Optimization, outcome, and rank	Model	AUROC^a^	*F*_1_-score	Sensitivity	PPV^b^
*F*_1_-score					
95th percentile cost					
1	XGBoost^c^	0.886^d^	0.411^d^	0.298	0.659
2	Random forest	0.851	0.401	0.436^d^	0.372
3	Logistic regression	0.886^d^	0.367	0.249	0.701
4	Reduced logistic regression	0.885	0.366	0.248	0.699
5	Elastic net	0.886^d^	0.356	0.239	0.703
6	LASSO^e^	0.885	0.351	0.234	0.707^d^
Hospitalization					
1	Logistic regression	0.841^d^	0.341^d^	0.233	0.631^d^
2	Reduced logistic regression	0.841^d^	0.340	0.233	0.630
3	Elastic net	0.840	0.339	0.232	0.630
4	LASSO	0.840	0.339	0.232	0.630
5	XGBoost	0.834	0.328	0.227	0.596
6	Random forest	0.811	0.299	0.276^d^	0.327
AUC^f^					
95th percentile cost					
1	XGBoost	0.891^d^	0.403^d^	0.282	0.706
2	Logistic regression	0.886	0.367	0.249	0.701
3	Elastic net	0.886	0.354	0.237	0.705
4	Reduced logistic regression	0.885	0.366	0.248	0.699
5	LASSO	0.885	0.351	0.234	0.707^d^
6	Random forest	0.850	0.272	0.754^d^	0.166
Hospitalization					
1	XGBoost	0.849^d^	0.321	0.209	0.689^d^
2	Elastic net	0.842	0.280	0.181	0.626
3	Logistic regression	0.841	0.341^d^	0.233	0.631
4	Reduced logistic regression	0.840	0.340	0.233	0.630
5	LASSO	0.840	0.339	0.232	0.630
6	Random forest	0.817	0.288	0.532^d^	0.198

a AUROC: area under receiver operating characteristic.

b PPV: positive predictive value.

c XGBoost: Extreme Gradient Boosting.

d Performance estimates for candidate models are based on the appropriate evaluation metric.

e LASSO: least absolute shrinkage and selection operator.

f AUC: area under the curve.

When parameters are fit to maximize AUROC, XGBoost is best suited for identifying prospective-year patients above the 95th percentile of cost (AUROC=0.891, 95% CI 0.891‐0.892) and prospective-year hospitalization (AUROC=0.849, 95% CI 0.848‐0.850). For both outcomes, the cross-validated AUROC of XGBoost is significantly different from the 95% CI of the corresponding logistic regressions for that task (95th percentile cost: 0.885‐0.886; hospitalization: 0.841‐0.842). Furthermore, average PPV is significantly better than regressions for the hospitalization task (PPV=0.689, 95% CI 0.687‐0.692). AUROC-optimized point estimates of *F*_1_ were again only significantly better for XGBoost in the prediction of high cost, not hospitalization.

On average, 132 of 268 features were retained by the *F*_1_-optimized elastic net model predicting hospitalization, 268 by the random forest, and 251 by XGBoost (Table 3). For *F*_1_-optimized models predicting the 95th percentile of cost, it was 191, 251, and 247 out of 251, respectively, suggesting there is a higher proportion of informative features estimating cost than hospitalization. This was consistent with observed AUROC-optimized selected features, though more were generally retained on average.

**Table 3.** Feature selection summary, reflecting the average integer count of selected features by model type, optimization, and outcome across cross-validations.

Optimization and model type	Hospitalization, n (%)	95th percentile cost, n (%)
*F*_1_-score		
Logistic regression	268 (100)	251 (100)
Elastic net	132 (49.3)	191 (76.1)
LASSO^a^	123 (45.9)	131 (52.2)
Random forest	268 (100)	251 (100)
Reduced logistic regression	123 (45.9)	131 (52.2)
XGBoost^b^	251 (93.7)	247 (98.4)
AUC^c^		
Logistic regression	268 (100)	251 (100)
Elastic net	268 (100)	164 (65.3)
LASSO	123 (45.9)	131 (52.2)
Random forest	268 (100)	251 (100)
Reduced logistic regression	123 (45.9)	131 (52.2)
XGBoost	254 (94.8)	237 (94.4)

a LASSO: least absolute shrinkage and selection operator.

b XGBoost: Extreme Gradient Boosting.

c AUC: area under the curve.

**Figure 2.**
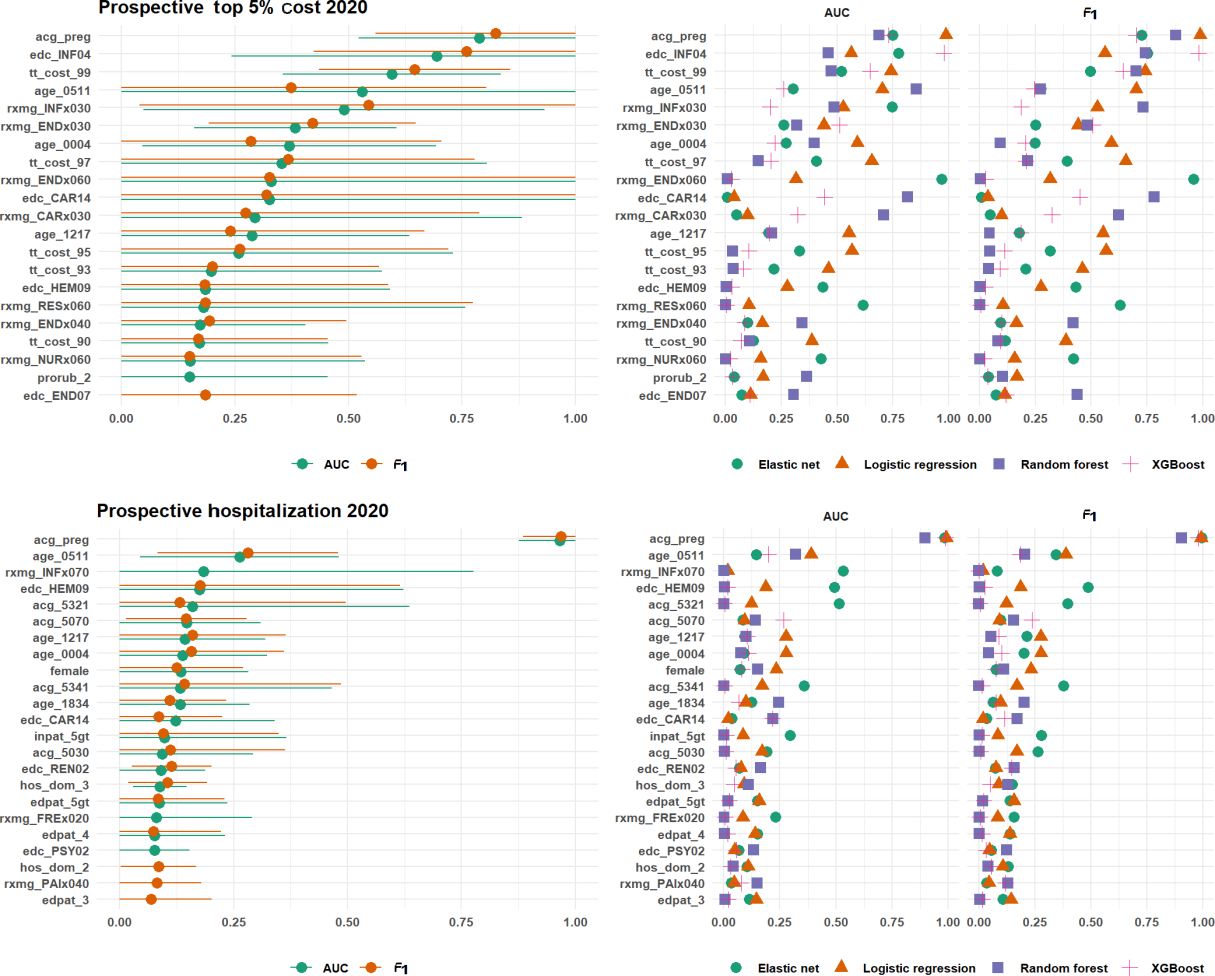
Average rescaled variable importance of the top 20 ranked features across all models for each optimization (left) and for each separate technique (right). The top 20 features were identified first through either optimization setting, meaning not all of the listed features were among the top-ranked. As a result, point traces may not appear for some features in the left panel, but all associated values are plotted on the right panel. AUC: area under the curve; XGBoost: Extreme Gradient Boosting.

Figure 2 illustrates the average variable importance and 95% CI for the top 20 ranked attributes for each task, across the performances of all 4 constituent models: logistic regression, elastic net, random forest, and XGBoost (left panel). Variable naming within the ACG System is detailed as part of an appended table and briefly described in Table S4 of Multimedia Appendix 1. Features ranking high in average importance, relative to all other features of the same model, are listed in descending order, and the average importance for each individual model is provided on the right panel of Figure 2. A great deal of variability is noted across the 4 models in terms of the order and degree of contribution for the top-ranked variables. For example, medications for endocrine disorders affecting growth (rxmg_ENDx060) are the most important feature for determining prospective-year elevated cost (95th percentile), but only in the elastic net models.

Features most strongly associated with elevated prospective-year total cost (at or above the 95th percentile) include pregnancy and maternity conditions (acg_preg), HIV or AIDS (edc_INF04), and concurrent-year total cost in the 98th to 99th percentile (tt_cost_99). For both *F*_1_- and AUROC-optimized models, the importance of pregnancy conditions is ranked first only for logistic regression, plausibly overstating its importance.

## Discussion

### Summary of Findings

ML techniques were found to significantly improve model sensitivity for the selected tasks. The *F*_1_-score for elevated prospective-year total cost was robustly improved by 12.0% over logistic regression by relying on an optimized XGBoost, but AUROC was improved only by 0.6%. For prospective-year hospitalization, these improvements were 0% and 1.0%, respectively. The performance estimates were consistent with those observed in prior research. When AUROC was optimized, it reached 0.849 and 0.891 for hospitalization and cost, respectively. Using the ACG score as a pretrained model, we have previously seen an AUROC of 0.761 for prospective-year hospitalization and 0.840 for elevated cost, within a cohort of 12,820 patients aged from 21 to 64 years from the same health system [35].

These findings may seem as though ML made little difference over regression, but these techniques also elevated the average point-estimated sensitivity by 0.033 (13.3%) and *F*_1_-score by 0.036 (9.8%), which calls into question the reliability of only inspecting the receiver operating characteristic. This finding meant better sensitivity without losing precision, relative to a regression model. Better sensitivity means more opportunities for detection and screening for poor health outcomes and better patient support.

Our results also illustrate how the use of ML optimization may only modestly improve precision and specificity over logistic regression. Whether enhanced sensitivity is worth the investment of time and resources for model training depends on the associated costs of each type of error; thus, the rationale for choosing one model over another does not boil down to a single metric. Regression offers clear advantages that may be more valuable to researchers, care administrators, and clinicians. The ability to dissect individual effects, such as shifting the odds of a predicted outcome, is powerful and grants care analysts meaningful insights into systematic bias that might be present in any model. This remains challenging, if not impossible, to achieve with most ML and advanced AI techniques.

### Significance

Several important points follow from these findings. For real-world tasks, it is generally not the case that there is a single best-performing model. Multiple acceptable solutions are considered, each with different strengths and weaknesses. A model with highly interpretable features (ie, regression) may be preferred over one that has better performance. Proper evaluation of model performance depends on understanding real-world costs of intervention. When these models serve as a decision support tool, the balance of precision and recall can only be properly calibrated after accounting for the cost of care and harm to patients.

Evaluating model performance is further nuanced when an outcome is severely imbalanced or skewed, as is often the case in real-world data. Our findings show that AUROC alone does not indicate which model should be used or what types of errors might be effectively reduced. The flexibility of ML approaches to fit specific use cases is generally their main advantage over the rigid statistical estimation found in regression. This is critical when an intervention is already in mind, and health systems are looking for decision support in administering care.

Consider a hypothetical program to screen patients with cardiovascular disease for unplanned hospital readmissions. From a denominator of 2000 patients with cardiovascular disease with hospital discharges annually, the program can afford to screen about 500. A statistical model that is calibrated to be overly precise might miss readmissions among those with less than high risk. Readmission is a rare event, but because the task requires identification of roughly 25% of the sample, there is a premium on model sensitivity over precision. In other words, the odds of a false negative (undetected readmission) are greater than false positives (cost of screening), so the selection of an appropriate model needs to account for this.

The *F*-beta statistic aids selection by weighting these preferences without prior knowledge of monetary or efficiency costs. The *F*_2_ statistic weighs sensitivity twice over precision, for example, and permits users to index average performance to suit their needs. Returning to our results, we can see how random forest models are suddenly much more appealing with tasks that require greater sensitivity without necessarily diminishing precision, as we would expect from simply shifting the classification threshold (Table S5 in Multimedia Appendix 1).

Lastly, several ML techniques are well-suited to feature selection, but there are sometimes disagreements on selected features and their relative importance. Our results show that elastic net is a powerful example of automated feature selection and maintains reasonable overall performance. We did not find that any ML technique is necessarily preferable over regression for interpretability, however. Some methods make it easier to understand which features are important for prediction, but several highly esteemed methods in the literature continue to be a black box, especially deep learning networks [36]. In practice, we see that it is often best to fit multiple types of models, with some minimum specification of performance in mind, and then select candidate models based on these practical constraints: performance, interpretability, and parsimony.

### Limitations

Our work is also limited in a variety of ways. The selected cohort may not be suitable for generalizable inference at a larger scale, as it primarily comprised Medicaid beneficiaries in the state of Maryland. In our prior validation efforts with earlier years of these data, we concluded that the rates of disease conditions and care utilization are not substantially different. We know social needs are more prevalent for this sample, and consequently, there may be a general elevation in health care utilization across most points of care, including hospitalization. Additionally, the timeframe overlaps with the beginning of the COVID-19 pandemic. This adds noise to our prospective year outcomes, and it is known that overall health care utilization was reduced during the early weeks and months especially, although rates of all-cause hospitalization were also likely more reflective of unplanned admissions for this year [37]. Finally, we did not perform a fully comprehensive grid search due to limitations in compute resources and the timeliness of providing results. Instead, a handful of impactful hyperparameters were varied for each technique across a broad range of values. Added performance is still possible with better refinement of these settings and further validated in this work with summary of CIs (Tables S6 and S7 in Multimedia Appendix 1).

Due to these limitations, the selected models require additional work to demonstrate their suitability for real-world use cases. This is especially true in the context of claims adjudication, where substantial financial harm could result from poorly calibrated models or those working based on erroneous assumptions, such as normality and homoscedasticity of residuals. The aim in this work is to first demonstrate the added predictive value of a cross-section of techniques but not attempt to exhaustively search all parameter settings as part of our grid search or prescribe clinical use cases. A more expansive approach, using more current, nationally representative data, is needed to validate these findings. The resulting cost and fairness of using these tools in a diversity of conditions also need to be assessed to ensure safety in implementation.

### Conclusions

Risk estimation can be improved somewhat by ML techniques over logistic regression, but there are several practical limitations. Unlike regression, ML techniques require parameter tuning and have more opaque variable interactions, making them far less interpretable. They may add parsimony to prediction efforts through feature selection, but often at the cost of performance. Conversely, the ability to tune parameters enables ML models to better tailor a response to fit specific use cases, for example, when greater precision or sensitivity is required, especially in imbalanced or skewed data. Overall improvement in precision and recall (separately or as the *F*_1_-score statistic) suggests decision tree ensembles are better suited to predicting imbalanced outcomes than logistic regression. This finding must be tempered by the observation that the gain in performance was also modest overall.

## Supplementary material

10.2196/92820 Multimedia Appendix 1 Inventories of model parameter settings, patient characteristics by patient need group, full CIs around cross-validated performance, and supporting descriptions of variables depicted in Figure 2.

10.2196/92820 Checklist 1 STROBE checklist.

## Abbreviations

ACG: Adjusted Clinical Group
AI: artificial intelligence
AUROC: area under the receiver operating characteristic
JHHP: Johns Hopkins Health Plans
LASSO: least absolute shrinkage and selection operator
ML: machine learning
PNG: patient need group
PPV: positive predictive value
STROBE: Strengthening the Reporting of Observational Studies in Epidemiology
XGBoost: Extreme Gradient Boosting
