# Supplementary material for: Improving Models to Predict Care Utilization Using Machine Learning: Retrospective Observational Study
Source: JMIR Form Res. 2026 Jun 26;10:e92820. doi: 10.2196/92820 (PMC13308755; doi:10.2196/92820)
Supplement: Multimedia Appendix 1 [file formative-v10-e92820-s001.docx]

**SUPPLEMENTAL MATERIALS**

Table S1. Optimization settings for each cross-validation task.

| Optimization | Outcome | Model | Final settings |
| --- | --- | --- | --- |
| AUROC | Hospitalization | Elastic net | {alpha: 0.0} |
|  |  | Random forest | {classwt: 1.5; depth: 130} |
|  |  | XGBoost | {eta: 0.1; depth: 10} |
|  | 95^th^ %tile total cost | Elastic net | {alpha: 0.2} |
|  |  | Random forest | {classwt: 0.8, depth: 130} |
|  |  | XGBoost | {eta: 0.1 depth: 10} |
| F1 | Hospitalization | Elastic net | {alpha: 0.5} |
|  |  | Random forest | {classwt: 2.5; depth: 130} |
|  |  | XGBoost | {eta: 1.0; depth: 4} |
|  | 95^th^ %tile total cost | Elastic net | {alpha: 0.1} |
|  |  | Random forest | {classwt: 2.5, depth: 140} |
|  |  | XGBoost | {eta: 0.3 depth: 10} |

Table S2. Study sample characteristics cross-tabulated by ACG Patient Need Groups (PNGs) from the first year (2019).*

| Variable | Non-User | Low Need Child | Low Need Adult | Multi-Morbidity Low Complexity | Multi-Morbidity Medium Complexity | Pregnancy Low Complexity | Pregnancy High Complexity | Dominant Psychiatric/Behavioral Condition | Dominant Major Chronic Condition | Multi-Morbidity High Complexity | Frailty | Total |
| --- | --- | --- | --- | --- | --- | --- | --- | --- | --- | --- | --- | --- |
| Total | 37,616 | 78,945 | 38,160 | 73,620 | 49,472 | 5,765 | 2,430 | 23,708 | 25,434 | 14,321 | 992 | 350,463 |
| Average age | 23.2 (15.8) | 7.6 (4.7) | 32.6 (13.1) | 21.9 (19.6) | 29.5 (23.8) | 26.8 (6.2) | 27.6 (6.2) | 31.2 (16.3) | 40.2 (20.6) | 42.9 (23.0) | 76.3 (7.5) | 24.1 (20.0) |
| Age 0-17 | 16,305 (43.3%) | 78,945 (100%) | 2,586 (6.8%) | 38,586 (52.4%) | 21,453 (43.4%) | 295 (5.1%) | 87 (3.6%) | 5,747 (24.2%) | 4,084 (16.1%) | 2,611 (18.2%) | 0 (0%) | 170,699 (48.7%) |
| Age 18-34 | 12,864 (34.2%) | 0 (0%) | 21,631 (56.7%) | 15,956 (21.7%) | 7,422 (15%) | 4,799 (83.2%) | 1,997 (82.2%) | 9,091 (38.3%) | 5,603 (22%) | 1,446 (10.1%) | 0 (0%) | 80,809 (23.1%) |
| Age 35-64 | 8,103 (21.5%) | 0 (0%) | 13,182 (34.5%) | 16,831 (22.9%) | 16,008 (32.4%) | 671 (11.6%) | 346 (14.2%) | 8,137 (34.3%) | 12,995 (51.1%) | 8,356 (58.3%) | 24 (2.4%) | 84,653 (24.2%) |
| Age 65+ | 344 (0.9%) | 0 (0%) | 761 (2%) | 2,247 (3.1%) | 4,589 (9.3%) | 0 (0%) | 0 (0%) | 733 (3.1%) | 2,752 (10.8%) | 1,908 (13.3%) | 968 (97.6%) | 14,302 (4.1%) |
| Female | 15,994 (42.5%) | 38932 (49.3%) | 21,207 (55.6%) | 42,440 (57.6%) | 28,178 (57%) | 5,765 (100%) | 2,430 (100%) | 15,070 (63.6%) | 15,559 (61.2%) | 7,778 (54.3%) | 631 (63.6%) | 193,984 (55.4%) |
| Male | 21,622 (57.5%) | 40013 (50.7%) | 16,953 (44.4%) | 31,180 (42.4%) | 21,294 (43%) | 0 (0%) | 0 (0%) | 8,638 (36.4%) | 9,875 (38.8%) | 6,543 (45.7%) | 361 (36.4%) | 156,479 (44.6%) |
| 1+ chronic condition | 0 (0%) | 14660 (18.6%) | 3,830 (10%) | 39,905 (54.2%) | 43,864 (88.7%) | 2,075 (36%) | 1,775 (73%) | 20,184 (85.1%) | 23,527 (92.5%) | 13,725 (95.8%) | 991 (99.9%) | 164,536 (46.9%) |
| 1+ active ingredient | 1,044 (2.8%) | 41800 (52.9%) | 25,361 (66.5%) | 59,583 (80.9%) | 44,734 (90.4%) | 4,762 (82.6%) | 2,349 (96.7%) | 22,428 (94.6%) | 23,993 (94.3%) | 13,510 (94.3%) | 964 (97.2%) | 240,528 (68.6%) |
| 1+ hospital stay | 0 (0%) | 74 (0.1%) | 23 (0.1%) | 2,869 (3.9%) | 2,993 (6%) | 2,364 (41%) | 1,489 (61.3%) | 1,475 (6.2%) | 3,409 (13.4%) | 6,911 (48.3%) | 468 (47.2%) | 22,075 (6.3%) |
| 95th %tile of cost | 0 (0%) | 53 (0.1%) | 70 (0.2%) | 512 (0.7%) | 1,678 (3.4%) | 1,243 (21.6%) | 1,042 (42.9%) | 1,314 (5.5%) | 5,160 (20.3%) | 5,959 (41.6%) | 493 (49.7%) | 17,524 (5%) |

* Patient need groups (PNGs) are groupings of the ACG risk adjustment system that characterizes 2019 patient care utilization and chronic disease comorbidity.

Table S3. Full performance metrics for binary outcome tasks with 95% CIs

|  |  | F1-Optimized | | | | AUROC-Optimized | | | |
| --- | --- | --- | --- | --- | --- | --- | --- | --- | --- |
| **Outcome** | **Model** | **AUROC** | **F1** | **Sensitivity** | **PPV** | **AUROC** | **F1** | **Sensitivity** | **PPV** |
| 95th %tile cost | Logistic regression | 0.886 (0.885:0.886) | 0.367 (0.366:0.369) | 0.249 (0.247:0.25) | 0.701 (0.699:0.704) | 0.886 (0.886:0.886) | 0.367 (0.366:0.369) | 0.249 (0.247:0.25) | 0.701 (0.699:0.703) |
|  | Elastic net | 0.886 (0.885:0.886) | 0.356 (0.354:0.358) | 0.239 (0.237:0.24) | 0.703 (0.7:0.706) | 0.886 (0.885:0.886) | 0.354 (0.353:0.356) | 0.237 (0.235:0.238) | 0.705 (0.702:0.707) |
|  | LASSO | 0.885 (0.885:0.886) | 0.351 (0.349:0.352) | 0.234 (0.232:0.235) | 0.707 (0.704:0.709) | 0.885 (0.885:0.886) | 0.351 (0.349:0.352) | 0.234 (0.232:0.235) | 0.707 (0.704:0.709) |
|  | Random forest | 0.851 (0.85:0.852) | 0.401 (0.4:0.403) | 0.436 (0.434:0.438) | 0.372 (0.371:0.374) | 0.85 (0.85:0.851) | 0.272 (0.271:0.274) | 0.754 (0.751:0.756) | 0.166 (0.165:0.167) |
|  | Reduced logistic regression | 0.885 (0.885:0.886) | 0.366 (0.364:0.367) | 0.248 (0.246:0.249) | 0.699 (0.697:0.702) | 0.885 (0.885:0.886) | 0.366 (0.364:0.367) | 0.248 (0.246:0.249) | 0.699 (0.697:0.702) |
|  | XGBoost | 0.886 (0.885:0.886) | 0.411 (0.409:0.412) | 0.298 (0.297:0.3) | 0.659 (0.657:0.662) | 0.891 (0.891:0.892) | 0.403 (0.401:0.404) | 0.282 (0.28:0.283) | 0.706 (0.704:0.709) |
| Hospitalization | Logistic regression | 0.841 (0.841:0.842) | 0.341 (0.339:0.342) | 0.233 (0.232:0.235) | 0.631 (0.628:0.634) | 0.841 (0.841:0.842) | 0.341 (0.339:0.343) | 0.233 (0.232:0.235) | 0.631 (0.629:0.634) |
|  | Elastic net | 0.84 (0.84:0.841) | 0.339 (0.337:0.341) | 0.232 (0.23:0.233) | 0.63 (0.628:0.633) | 0.842 (0.841:0.842) | 0.28 (0.277:0.284) | 0.181 (0.178:0.184) | 0.626 (0.623:0.629) |
|  | LASSO | 0.84 (0.839:0.84) | 0.339 (0.337:0.34) | 0.232 (0.23:0.233) | 0.63 (0.627:0.632) | 0.84 (0.839:0.84) | 0.339 (0.337:0.34) | 0.232 (0.23:0.233) | 0.63 (0.627:0.632) |
|  | Random forest | 0.811 (0.81:0.812) | 0.299 (0.295:0.303) | 0.276 (0.271:0.28) | 0.327 (0.323:0.33) | 0.817 (0.817:0.818) | 0.288 (0.287:0.29) | 0.532 (0.529:0.535) | 0.198 (0.196:0.199) |
|  | Reduced logistic regression | 0.841 (0.84:0.841) | 0.34 (0.339:0.342) | 0.233 (0.232:0.235) | 0.63 (0.628:0.633) | 0.841 (0.84:0.841) | 0.34 (0.339:0.342) | 0.233 (0.232:0.235) | 0.63 (0.628:0.633) |
|  | XGBoost | 0.834 (0.833:0.835) | 0.328 (0.327:0.33) | 0.227 (0.225:0.228) | 0.596 (0.593:0.598) | 0.849 (0.848:0.85) | 0.321 (0.319:0.322) | 0.209 (0.207:0.21) | 0.689 (0.687:0.692) |

Table S4. Short description of each of the top 20 features of importance across all models, for both prospective year hospitalization and elevated cost.

| Short name | Outcome | Description |
| --- | --- | --- |
| acg_preg | Hospitalization; cost | Indicator for Pregnancy and prenatal care |
| age_0511 | Hospitalization; cost | Age category for pts between 5-11 yrs |
| age_0004 | Hospitalization; cost | Age category 0-4 yrs |
| age_1217 | Hospitalization; cost | Age category 12-17 yrs |
| female | Hospitalization | Enrollee sex is Female |
| edc_HEM09 | Hospitalization; cost | Hematologic conditions like |
| acg_5030 | Hospitalization | ACG category capturing comorbidity profile of patients with 1-17 yrs age, more than 10 comorbidity conditions of which 2 are major comorbidity conditions. |
| acg_5341 | Hospitalization | ACG category capturing comorbidity profile of patients who are infants, low-birth weight, have at least 6 comorbidity conditions of which at least 1 is major comorbidity condition |
| edpat_5gt | Hospitalization | Count of emergency room visits (>5) that did not lead to a subsequent inpatient hospitalization |
| acg_4620 | Hospitalization | ACG category capturing comorbidity profile of patients who are 6-17 yrs age, have 6-9 comorbidity conditions of which at least one is major comorbidity condition. |
| edpat_3 | Hospitalization |  |
| acg_5342 | Hospitalization | ACG category capturing comorbidity profile of patients who are infants, normal birth weight, have at least 6 comorbidity conditions of which at least 1 is major comorbidity condition |
| acg_5020 | Hospitalization | ACG category capturing comorbidity profile of patients with 1-17 yrs age, more than 10 comorbidity conditions of which one is major comorbidity conditions. |
| edpat_4 | Hospitalization | Count of emergency room visits (4) that did not lead to a subsequent inpatient hospitalization |
| edpat_2 | Hospitalization | Count of emergency room visits (2) that did not lead to a subsequent inpatient hospitalization |
| rxmg_FREx011 | Hospitalization | Presence of drugs used for female reproductive system such as contraception meds. |
| acg_5321 | Hospitalization | ACG category capturing comorbidity profile of patients who are infants, low-birth weight, have 0-5 comorbidity conditions of which at least 1 is major comorbidity condition |
| acg_5322 | Hospitalization | ACG category capturing comorbidity profile of patients who are infants, normal birth weight, have 0-5 comorbidity conditions of which at least 1 is major comorbidity condition |
| hos_dom_2 | Hospitalization | Marker to identify hospital dominant conditions which are at high-risk of hospitalization in the subsequent year. |
| tt_cost_99 | Cost | Prior year total cost percentile – 99^th^ |
| tt_cost_97 | Cost | Prior year total cost percentile – 97^th^ |
| tt_cost_95 | Cost | Prior year total cost percentile – 95^th^ |
| edc_INF0O4 | Cost | Infection conditions such as HIV, AIDS |
| rxmg_INFx030 | Cost | Presence of drugs related to infection condition such as … |
| tt_cost_95 | Cost |  |
| rxmg_ENDx030 | Cost | Presence of drugs used for metabolic conditions such as diabetes e.g. insulin |
| tt_cost_90 | Cost | Prior year total cost percentile – 90^th^ |
| rxmg_ENDx060 | Cost | Presence of drugs used for endocrine/metabolic conditions such as growth problems. |
| tt_cost_75 | Cost | Prior year total cost percentile – 75^th^ |
| edc_GAS15 | Cost | Gastro-intestinal conditions such as hepatitis C |
| rxmg_SKNx030 | Cost | Presence of drugs used for chronic skin conditions |
| edc_GAS02 | Cost | Gastrointestinal conditions such as inflammatory bowel diseases |
| age_4554 | Cost | Age 45-54 yrs |
| tt_cost_50 | Cost | Prior year total cost percentile – 50^th^ |

Table S. F-beta point performances, spanning betas 0.333 to 3 and assuming a decision threshold of response at p(x)>0.5. Original rankings of performance is preserved from Table 2 and betas selected only to illustrate relative best performance (bold and underlined font) when cost calibrations require better precision (beta < 1) or sensitivity (beta > 1).

|  |  |  |  | **More Precise<---------Equal--------->More Sensitive** | | | | |
| --- | --- | --- | --- | --- | --- | --- | --- | --- |
| **Opt** | **Outcome** | **Rank** | **Model** | **F0.333** | **F0.5** | **F1** | **F2** | **F3** |
| F1 | 95th %tile cost | 1 | XGBoost | 0.588 | **0.530** | **0.411** | 0.335 | 0.315 |
|  |  | 2 | Random forest | 0.378 | 0.384 | 0.402 | **0.421** | **0.428** |
|  |  | 3 | Logistic regression | **0.592** | 0.513 | 0.367 | 0.285 | 0.265 |
|  |  | 4 | Reduced logistic regression | 0.592 | 0.512 | 0.365 | 0.284 | 0.264 |
|  |  | 5 | Elastic net | 0.588 | 0.505 | 0.356 | 0.274 | 0.255 |
|  |  | 6 | LASSO | 0.588 | 0.503 | 0.351 | 0.269 | 0.250 |
|  | Hospitalization | 1 | Logistic regression | **0.540** | **0.471** | **0.341** | 0.267 | 0.249 |
|  |  | 2 | Reduced logistic regression | 0.539 | 0.470 | 0.34 | 0.267 | 0.249 |
|  |  | 3 | Elastic net | 0.538 | 0.469 | 0.339 | 0.266 | 0.248 |
|  |  | 4 | LASSO | 0.537 | 0.468 | 0.338 | 0.265 | 0.247 |
|  |  | 5 | XGBoost | 0.511 | 0.448 | 0.329 | 0.259 | 0.242 |
|  |  | 6 | Random forest | 0.326 | 0.321 | 0.305 | **0.292** | **0.287** |
| **Opt** | **Outcome** | **Rank** | **Model** | **F0.333** | **F0.5** | **F1** | **F2** | **F3** |
| AUC | 95th %tile cost | 1 | XGBoost | **0.614** | **0.543** | **0.402** | 0.320 | 0.299 |
|  |  | 2 | Logistic regression | 0.593 | 0.514 | 0.367 | 0.286 | 0.266 |
|  |  | 3 | Elastic net | 0.592 | 0.513 | 0.365 | 0.284 | 0.264 |
|  |  | 4 | Reduced logistic regression | 0.592 | 0.512 | 0.365 | 0.284 | 0.264 |
|  |  | 5 | LASSO | 0.588 | 0.503 | 0.351 | 0.269 | 0.250 |
|  |  | 6 | Random forest | 0.181 | 0.198 | 0.274 | **0.442** | **0.556** |
|  | Hospitalization | 1 | XGBoost | **0.561** | **0.473** | 0.321 | 0.243 | 0.225 |
|  |  | 2 | Elastic net | 0.524 | 0.447 | 0.31 | 0.238 | 0.221 |
|  |  | 3 | Logistic regression | 0.540 | 0.472 | **0.341** | 0.268 | 0.250 |
|  |  | 4 | Reduced logistic regression | 0.538 | 0.470 | 0.34 | 0.267 | 0.249 |
|  |  | 5 | LASSO | 0.537 | 0.468 | 0.338 | 0.265 | 0.247 |
|  |  | 6 | Random forest | 0.210 | 0.225 | 0.288 | **0.397** | **0.455** |

Table S6. Average variable importance estimates and 95% confidence intervals, scaled 0 to 1 on the top 20 predictors for prospective year hospitalization across all models.

| Hosp. | AUC | | | | | | F1 | | | | | |
| --- | --- | --- | --- | --- | --- | --- | --- | --- | --- | --- | --- | --- |
| Coefficient | Regression | Elastic net | LASSO | Random forest | Regularized regression | xgboost | Regression | Elastic net | LASSO | Random forest | Regularized regression | xgboost |
| acg_preg | 0.994 (0.983:1) | 0.985 (0.959:1) | 0.994 (0.983:1) | 0.897 (0.722:1) | 0.982 (0.934:1) | 0.988 (0.966:1) | 0.994 (0.982:1) | 0.995 (0.984:1) | 0.994 (0.983:1) | 0.905 (0.733:1) | 0.982 (0.934:1) | 0.98 (0.928:1) |
| age_0511 | 0.389 (0.378:0.399) | 0.144 (0.115:0.173) | 0.392 (0.36:0.424) | 0.319 (0.216:0.422) | 0.329 (0.323:0.334) | 0.199 (0.186:0.213) | 0.388 (0.378:0.398) | 0.346 (0.314:0.377) | 0.392 (0.36:0.424) | 0.205 (0.142:0.268) | 0.329 (0.323:0.334) | 0.185 (0.135:0.235) |
| age_0004 | 0.278 (0.268:0.289) | 0.091 (0.073:0.11) | 0.228 (0.198:0.259) | 0.075 (0.042:0.109) | 0.238 (0.228:0.248) | 0.108 (0.101:0.115) | 0.278 (0.268:0.288) | 0.202 (0.174:0.231) | 0.228 (0.198:0.259) | 0.044 (0.024:0.064) | 0.238 (0.228:0.248) | 0.101 (0.058:0.144) |
| age_1217 | 0.278 (0.266:0.289) | 0.092 (0.077:0.107) | 0.243 (0.219:0.267) | 0.1 (0.06:0.14) | 0.246 (0.238:0.253) | 0.104 (0.096:0.112) | 0.277 (0.267:0.288) | 0.215 (0.191:0.24) | 0.243 (0.219:0.267) | 0.055 (0.032:0.077) | 0.246 (0.238:0.253) | 0.089 (0.055:0.122) |
| female | 0.234 (0.22:0.247) | 0.072 (0.063:0.081) | 0.074 (0.064:0.084) | 0.152 (0.102:0.203) | 0.174 (0.162:0.185) | 0.08 (0.073:0.087) | 0.234 (0.22:0.248) | 0.076 (0.067:0.085) | 0.074 (0.064:0.084) | 0.112 (0.073:0.152) | 0.174 (0.162:0.185) | 0.076 (0.041:0.111) |
| edc_HEM09 | 0.187 (0.168:0.206) | 0.493 (0.448:0.539) | 0.487 (0.439:0.535) | 0.003 (0.001:0.005) | 0.154 (0.141:0.168) | 0.017 (0.013:0.02) | 0.187 (0.17:0.203) | 0.489 (0.447:0.53) | 0.487 (0.439:0.535) | 0.003 (0.001:0.005) | 0.154 (0.141:0.168) | 0.026 (0.01:0.042) |
| acg_5030 | 0.171 (0.15:0.192) | 0.192 (0.146:0.237) | 0.278 (0.217:0.339) | 0.002 (0.001:0.004) | 0.127 (0.11:0.144) | 0.009 (0.004:0.015) | 0.171 (0.151:0.191) | 0.265 (0.203:0.326) | 0.278 (0.217:0.339) | 0.001 (0:0.003) | 0.127 (0.11:0.144) | 0.008 (0:0.019) |
| acg_5341 | 0.171 (0.149:0.192) | 0.358 (0.301:0.415) | 0.38 (0.299:0.461) | 0.001 (0:0.002) | 0.125 (0.106:0.143) | 0.003 (0.001:0.004) | 0.171 (0.15:0.192) | 0.378 (0.299:0.457) | 0.38 (0.299:0.461) | 0 (0:0.001) | 0.125 (0.106:0.143) | 0.015 (0:0.056) |
| edpat_5gt | 0.157 (0.14:0.174) | 0.148 (0.133:0.164) | 0.139 (0.116:0.163) | 0.017 (0.008:0.026) | 0.122 (0.108:0.135) | 0.024 (0.02:0.027) | 0.157 (0.139:0.174) | 0.14 (0.121:0.16) | 0.139 (0.116:0.163) | 0.019 (0.009:0.028) | 0.122 (0.108:0.135) | 0.022 (0.011:0.032) |
| acg_4620 | 0.148 (0.132:0.165) | 0.057 (0.027:0.086) | 0.066 (0:0.133) | 0.001 (0:0.001) | 0.104 (0.09:0.118) | 0.007 (0.004:0.009) | 0.148 (0.132:0.165) | 0.061 (0:0.125) | 0.066 (0:0.133) | 0 (0:0.001) | 0.104 (0.09:0.118) | 0.004 (0:0.008) |
| edpat_3 | 0.145 (0.129:0.16) | 0.113 (0.098:0.128) | 0.105 (0.085:0.126) | 0.007 (0.002:0.011) | 0.114 (0.101:0.128) | 0.02 (0.016:0.023) | 0.145 (0.128:0.161) | 0.107 (0.088:0.125) | 0.105 (0.085:0.126) | 0.006 (0.002:0.01) | 0.114 (0.101:0.128) | 0.016 (0.009:0.023) |
| acg_5342 | 0.141 (0.122:0.16) | 0.136 (0.092:0.179) | 0.108 (0.025:0.19) | 0 (0:0.001) | 0.095 (0.08:0.11) | 0.003 (0.002:0.005) | 0.141 (0.123:0.159) | 0.111 (0.029:0.193) | 0.108 (0.025:0.19) | 0 (0:0) | 0.095 (0.08:0.11) | 0.006 (0:0.016) |
| edpat_4 | 0.139 (0.124:0.155) | 0.15 (0.131:0.17) | 0.138 (0.115:0.161) | 0.002 (0.001:0.004) | 0.109 (0.098:0.121) | 0.016 (0.013:0.018) | 0.139 (0.123:0.155) | 0.14 (0.117:0.163) | 0.138 (0.115:0.161) | 0.002 (0.001:0.004) | 0.109 (0.098:0.121) | 0.014 (0.009:0.02) |
| acg_5020 | 0.134 (0.116:0.152) | 0.1 (0.06:0.14) | 0.094 (0.016:0.173) | 0.001 (0:0.001) | 0.091 (0.077:0.104) | 0.004 (0.002:0.006) | 0.134 (0.112:0.155) | 0.089 (0.009:0.17) | 0.094 (0.016:0.173) | 0 (0:0.001) | 0.091 (0.077:0.104) | 0.004 (0:0.008) |
| edpat_2 | 0.131 (0.115:0.146) | 0.07 (0.06:0.08) | 0.061 (0.048:0.075) | 0.004 (0.001:0.007) | 0.105 (0.093:0.117) | 0.019 (0.016:0.022) | 0.131 (0.116:0.146) | 0.063 (0.049:0.076) | 0.061 (0.048:0.075) | 0.004 (0.001:0.006) | 0.105 (0.093:0.117) | 0.014 (0.008:0.021) |
| rxmg_FREx011 | 0.125 (0.112:0.139) | 0.032 (0.018:0.045) | 0.043 (0.024:0.061) | 0.008 (0.003:0.014) | 0.099 (0.089:0.11) | 0.03 (0.027:0.033) | 0.125 (0.111:0.139) | 0.044 (0.027:0.061) | 0.043 (0.024:0.061) | 0.007 (0.002:0.012) | 0.099 (0.089:0.11) | 0.028 (0.023:0.034) |
| acg_5321 | 0.123 (0.094:0.152) | 0.514 (0.395:0.633) | 0.376 (0.167:0.586) | 0 (0:0) | 0.087 (0.068:0.106) | 0.003 (0.001:0.004) | 0.123 (0.096:0.15) | 0.397 (0.21:0.584) | 0.376 (0.167:0.586) | 0 (0:0) | 0.087 (0.068:0.106) | 0.005 (0:0.013) |
| acg_5322 | 0.115 (0.094:0.136) | 0.165 (0.11:0.22) | 0.068 (0:0.146) | 0 (0:0) | 0.078 (0.059:0.097) | 0.003 (0.002:0.004) | 0.115 (0.093:0.138) | 0.083 (0:0.196) | 0.068 (0:0.146) | 0 (0:0) | 0.078 (0.059:0.097) | 0.008 (0:0.016) |
| hos_dom_2 | 0.108 (0.092:0.124) | 0.102 (0.09:0.114) | 0.136 (0.118:0.154) | 0.041 (0.021:0.062) | 0.085 (0.073:0.097) | 0.031 (0.025:0.037) | 0.108 (0.094:0.122) | 0.132 (0.117:0.146) | 0.136 (0.118:0.154) | 0.043 (0.023:0.062) | 0.085 (0.073:0.097) | 0.057 (0.015:0.099) |

Table S7. Average variable importance estimates and 95% confidence intervals, scaled 0 to 1 on the top 20 predictors for prospective year elevated cost across all models.

| Elevated Cost | AUC | | | | | | F1 | | | | | |
| --- | --- | --- | --- | --- | --- | --- | --- | --- | --- | --- | --- | --- |
| Coefficient | Regression | Elastic net | LASSO | Random forest | Regularized regression | xgboost | Regression | Elastic net | LASSO | Random forest | Regularized regression | xgboost |
| acg_preg | 0.988 (0.965:1) | 0.748 (0.71:0.786) | 0.749 (0.708:0.789) | 0.687 (0.494:0.88) | 0.99 (0.972:1) | 0.73 (0.703:0.757) | 0.988 (0.965:1) | 0.726 (0.691:0.761) | 0.749 (0.708:0.789) | 0.88 (0.677:1) | 0.99 (0.972:1) | 0.702 (0.671:0.733) |
| tt_cost_99 | 0.742 (0.715:0.768) | 0.52 (0.481:0.559) | 0.552 (0.519:0.585) | 0.473 (0.34:0.605) | 0.644 (0.621:0.666) | 0.648 (0.552:0.745) | 0.742 (0.716:0.768) | 0.497 (0.451:0.542) | 0.552 (0.519:0.585) | 0.702 (0.486:0.918) | 0.644 (0.621:0.666) | 0.643 (0.546:0.741) |
| age_0511 | 0.701 (0.684:0.718) | 0.301 (0.251:0.352) | 0.374 (0.327:0.421) | 0.855 (0.65:1) | 0.472 (0.464:0.48) | 0.261 (0.245:0.277) | 0.702 (0.684:0.719) | 0.274 (0.205:0.343) | 0.374 (0.327:0.421) | 0.274 (0.178:0.37) | 0.472 (0.464:0.48) | 0.245 (0.229:0.26) |
| tt_cost_97 | 0.655 (0.629:0.681) | 0.407 (0.371:0.444) | 0.43 (0.397:0.462) | 0.146 (0.091:0.201) | 0.567 (0.543:0.591) | 0.202 (0.182:0.222) | 0.655 (0.632:0.678) | 0.391 (0.346:0.435) | 0.43 (0.397:0.462) | 0.214 (0.14:0.288) | 0.567 (0.543:0.591) | 0.208 (0.187:0.229) |
| age_0004 | 0.591 (0.575:0.606) | 0.271 (0.218:0.325) | 0.328 (0.279:0.377) | 0.397 (0.259:0.535) | 0.416 (0.407:0.424) | 0.22 (0.206:0.234) | 0.591 (0.578:0.604) | 0.249 (0.179:0.318) | 0.328 (0.279:0.377) | 0.093 (0.047:0.139) | 0.416 (0.407:0.424) | 0.206 (0.187:0.225) |
| tt_cost_95 | 0.566 (0.542:0.591) | 0.33 (0.296:0.363) | 0.348 (0.315:0.381) | 0.033 (0.016:0.05) | 0.462 (0.442:0.482) | 0.105 (0.094:0.116) | 0.567 (0.542:0.592) | 0.316 (0.278:0.355) | 0.348 (0.315:0.381) | 0.047 (0.021:0.073) | 0.462 (0.442:0.482) | 0.111 (0.095:0.128) |
| edc_INF04 | 0.562 (0.536:0.588) | 0.775 (0.723:0.826) | 0.782 (0.728:0.836) | 0.461 (0.337:0.585) | 0.385 (0.365:0.405) | 0.978 (0.936:1) | 0.562 (0.536:0.588) | 0.754 (0.711:0.796) | 0.782 (0.728:0.836) | 0.742 (0.504:0.981) | 0.385 (0.365:0.405) | 0.981 (0.945:1) |
| age_1217 | 0.552 (0.534:0.57) | 0.193 (0.155:0.231) | 0.23 (0.196:0.263) | 0.207 (0.125:0.289) | 0.361 (0.351:0.372) | 0.195 (0.186:0.204) | 0.553 (0.536:0.569) | 0.177 (0.126:0.228) | 0.23 (0.196:0.263) | 0.044 (0.016:0.072) | 0.361 (0.351:0.372) | 0.184 (0.171:0.198) |
| rxmg_INFx030 | 0.527 (0.497:0.557) | 0.747 (0.696:0.799) | 0.745 (0.698:0.792) | 0.487 (0.308:0.666) | 0.37 (0.347:0.394) | 0.199 (0.169:0.229) | 0.527 (0.496:0.559) | 0.731 (0.68:0.781) | 0.745 (0.698:0.792) | 0.733 (0.539:0.927) | 0.37 (0.347:0.394) | 0.186 (0.164:0.208) |
| tt_cost_93 | 0.461 (0.436:0.485) | 0.217 (0.188:0.246) | 0.235 (0.21:0.26) | 0.033 (0.013:0.053) | 0.353 (0.335:0.37) | 0.08 (0.069:0.091) | 0.461 (0.437:0.484) | 0.206 (0.172:0.241) | 0.235 (0.21:0.26) | 0.041 (0.015:0.066) | 0.353 (0.335:0.37) | 0.092 (0.079:0.105) |
| rxmg_ENDx030 | 0.44 (0.417:0.464) | 0.261 (0.242:0.28) | 0.267 (0.25:0.285) | 0.318 (0.187:0.45) | 0.309 (0.293:0.325) | 0.511 (0.411:0.61) | 0.441 (0.417:0.464) | 0.251 (0.235:0.267) | 0.267 (0.25:0.285) | 0.482 (0.312:0.653) | 0.309 (0.293:0.325) | 0.507 (0.39:0.625) |
| tt_cost_90 | 0.386 (0.364:0.408) | 0.124 (0.1:0.147) | 0.14 (0.121:0.159) | 0.107 (0.059:0.155) | 0.312 (0.296:0.327) | 0.069 (0.06:0.078) | 0.387 (0.364:0.409) | 0.116 (0.088:0.144) | 0.14 (0.121:0.159) | 0.081 (0.044:0.118) | 0.312 (0.296:0.327) | 0.095 (0.08:0.109) |
| rxmg_ENDx060 | 0.316 (0.293:0.338) | 0.969 (0.903:1) | 0.962 (0.888:1) | 0.007 (0.002:0.012) | 0.228 (0.213:0.244) | 0.026 (0.021:0.031) | 0.316 (0.295:0.336) | 0.959 (0.881:1) | 0.962 (0.888:1) | 0.004 (0.001:0.008) | 0.228 (0.213:0.244) | 0.027 (0.022:0.032) |
| tt_cost_75 | 0.275 (0.251:0.298) | 0.025 (0.001:0.049) | 0.032 (0.01:0.054) | 0.049 (0.031:0.067) | 0.154 (0.14:0.168) | 0.044 (0.037:0.051) | 0.275 (0.253:0.296) | 0.024 (0:0.049) | 0.032 (0.01:0.054) | 0.051 (0.032:0.069) | 0.154 (0.14:0.168) | 0.062 (0.051:0.073) |
| edc_HEM09 | 0.275 (0.247:0.303) | 0.436 (0.389:0.483) | 0.425 (0.374:0.476) | 0.003 (0:0.005) | 0.192 (0.172:0.211) | 0.027 (0.022:0.031) | 0.275 (0.244:0.307) | 0.431 (0.385:0.477) | 0.425 (0.374:0.476) | 0.001 (0:0.003) | 0.192 (0.172:0.211) | 0.026 (0.021:0.031) |
| edc_GAS15 | 0.257 (0.229:0.285) | 0.192 (0.171:0.214) | 0.182 (0.16:0.204) | 0.02 (0.005:0.035) | 0.181 (0.165:0.197) | 0.055 (0.046:0.065) | 0.257 (0.232:0.282) | 0.193 (0.173:0.213) | 0.182 (0.16:0.204) | 0.04 (0.015:0.065) | 0.181 (0.165:0.197) | 0.06 (0.051:0.069) |
| rxmg_SKNx030 | 0.215 (0.191:0.239) | 0.319 (0.281:0.356) | 0.3 (0.26:0.34) | 0.001 (0:0.002) | 0.153 (0.135:0.171) | 0.019 (0.015:0.022) | 0.215 (0.187:0.243) | 0.319 (0.282:0.355) | 0.3 (0.26:0.34) | 0.002 (0:0.003) | 0.153 (0.135:0.171) | 0.019 (0.015:0.024) |
| edc_GAS02 | 0.2 (0.172:0.228) | 0.202 (0.175:0.229) | 0.191 (0.163:0.219) | 0.003 (0:0.007) | 0.135 (0.12:0.151) | 0.014 (0.009:0.019) | 0.2 (0.177:0.223) | 0.202 (0.178:0.226) | 0.191 (0.163:0.219) | 0.005 (0:0.009) | 0.135 (0.12:0.151) | 0.016 (0.011:0.022) |
| age_4554 | 0.194 (0.175:0.214) | 0.01 (0:0.021) | 0.01 (0:0.021) | 0.007 (0:0.015) | 0.086 (0.075:0.098) | 0.025 (0.021:0.029) | 0.195 (0.176:0.213) | 0.011 (0:0.023) | 0.01 (0:0.021) | 0.004 (0:0.01) | 0.086 (0.075:0.098) | 0.041 (0.03:0.051) |
| tt_cost_50 | 0.193 (0.17:0.216) | 0.014 (0.001:0.028) | 0.002 (0:0.006) | 0.158 (0.079:0.237) | - | 0.044 (0.035:0.052) | 0.193 (0.172:0.214) | 0.019 (0.005:0.034) | 0.002 (0:0.006) | 0.063 (0.037:0.089) | - | 0.052 (0.042:0.062) |
